# The Twilight Zone Where Margins Are Blurred: A Retrospective Analysis of Unilateral and Bilateral Optic Disc Swelling in a Tertiary Center in Malaysia

**DOI:** 10.7759/cureus.35823

**Published:** 2023-03-06

**Authors:** Shew Fei Chee, Chang Feng Chew, Embong Zunaina

**Affiliations:** 1 Department of Ophthalmology and Visual Science, School of Medical Sciences, Universiti Sains Malaysia, Kubang Kerian, MYS; 2 Department of Ophthalmology, Hospital Raja Permaisuri Bainun, Ipoh, MYS

**Keywords:** diabetic papillopathy, papilledema, non-arteritic anterior ischemic optic neuropathy, optic neuritis, bilateral optic disc swelling, unilateral optic disc swelling, optic disc swelling

## Abstract

Background

To analyze the etiology of optic disc swelling (ODS) and compare the clinical features between non-arteritic anterior ischemic optic neuropathy (NA-AION) and optic neuritis (ON) at our center from January 2019 to January 2020.

Methodology

Clinical records of all patients who presented with ODS between January 2019 and January 2020 were reviewed. The collected data were analyzed using IBM SPSS Statistics for Windows (Version 23.0, IBM Corp., Armonk, NY, USA).

Results

A total of 70 eyes among 47 patients were included in this review. There were 24 patients (51%) who had unilateral ODS, while 23 patients (49.0%) had bilateral ODS. The most common etiology of unilateral ODS was ON (45.7%), NA-AION (25%), and neuroretinitis (12.5%). Among bilateral ODS, the most common cause was papilledema (30.4%), hypertensive retinopathy (21.7%), diabetic papillitis (13.1%), and optic disc drusen (13.1%). Among unilateral ODS, the mean onset age was significantly older in NA-AION cases than that in ON cases (57.5 years vs. 40.3 years, *P *= 0.016). Ocular pain was a significant clinical finding observed in ON cases (54.5%) and none in NA-AION cases (*P *= 0.043). Although there was no significant difference in the initial visual acuity (VA) between NA-AION and ON, the final VA was significantly improved in ON compared to NA-AION (0.31 ± 0.34 vs. 1.14 ± 1.00, *P* = 0.029).

Conclusions

Many differential diagnoses must be considered when a patient is presented with ODS. With the increased prevalence of hypertension and diabetes in Malaysia, it is not a surprise that both hypertensive retinopathy and diabetic papillitis together accounted for one-third of the bilateral ODS patients.

## Introduction

Optic disc swelling (ODS), also known as optic disc edema, is a broad term for the ophthalmological finding of swelling of the optic nerve head [1]. Because this pathological condition has multiple etiologies, the ability to differentiate between them and arrive at a final diagnosis requires a comprehensive history and clinical examination.

The most common causes of unilateral ODS differ from those of bilateral ODS. Optic neuritis (ON), non-arteritic anterior ischemic optic neuropathy (NA-AION), and neuroretinitis are some of the most common conditions associated with unilateral ODS. Although ON and NA-AION can be one of the differential diagnoses for bilateral ODS, they should only be considered after other more common causes, such as increased intracranial pressure, hypertensive retinopathy, and diabetic papillitis, have been ruled out [2].

In Malaysia, there is only one published study on the clinical profile of unilateral and bilateral ODS [3]. Therefore, the goal of this study is to investigate the etiologies of unilateral and bilateral ODS and compare the clinical features of NA-AION and ON in our local setting.

## Materials and methods

The National Medical Research Register approved this study. We adhered to the tenets of the Declaration of Helsinki.

This was a retrospective study carried out at Hospital Raja Permaisuri Bainun in Malaysia. All patients who presented with ODS at the initial visit to Hospital Raja Perempuan Bainun in Malaysia between January 2019 and January 2020 had their clinical records of demographic data and clinical presentation reviewed. Patients who missed the subsequent three months of follow-up and those who first presented with optic disc pallor were excluded. We excluded patients who were referred to the on-call team or from the emergency department. Visual acuity (VA), red saturation, light brightness sensitivity, color vision, slit lamp examination, and Humphrey visual field examination were all performed on all patients. Fundus examinations were carried out using a 90-dioptre (D) lens. Fundus photographs were taken during the initial and subsequent visits to document the progression of the disease.

The following standardized criteria were used to determine the diagnosis of ON and NA-AION: the Optic Neuritis Treatment Trial (ONTT) criteria were used to define ON, and the Ischemic Optic Neuropathy Decompression Trial (IONDT) criteria were used to define NA-AION [4,5]. All ON and NA-AION patients, regardless of age, were included.

Papilledema was diagnosed when optic disc edema was secondary to increased intracranial pressure. Diabetic papillitis was defined by the presence of diabetes and optic disc edema in the absence of substantial optic nerve dysfunction, ocular inflammation, or elevated intracranial pressure [6]. Ophthalmological examinations and imaging tests both supported the diagnosis of compressive optic neuropathy. Other diseases, such as central retinal vein occlusion, were diagnosed based on their distinctive clinical features. VA at presentation was determined as the initial VA, while VA at one-year follow-up was determined as the final VA.

The collected data were analyzed using Microsoft Excel and SPSS Version 23 (IBM Corp., Armonk, NY, USA). Numerical data were analyzed using the Mann-Whitney test. For categorical variables, Fisher’s exact test was used. A *P*-value of less than 0.05 was considered to be statistically significant.

## Results

This review included a total of 70 eyes from 47 patients. There were 24 patients with unilateral ODS (51.0%) and 23 patients with bilateral ODS (49.0%).

ON (45.7%) was the most common cause of unilateral ODS, followed by NA-AION (25.0%) and neuroretinitis (12.5%). Other causes of unilateral ODS were optic disc drusen (4.2%), infiltrative optic neuropathy (4.2%), central retinal vein occlusion (CRVO) (4.2%), and compressive optic neuropathy (4.2%) (Table 1).

**Table 1:** Percentage distribution of unilateral and bilateral ODS. ODS, optic disc swelling

Etiology	Unilateral ODS (*n* = 24 patients), *n* (%)	Bilateral ODS (*n* = 23 patients), *n* (%)
Papilledema	0	7 (30.4)
Hypertensive retinopathy	0	5 (21.7)
Diabetic papillitis	0	3 (13.1)
Optic disc drusen	1 (4.2)	3 (13.1)
Infiltrative optic neuropathy	1 (4.2)	2 (8.7)
Optic neuritis	11 (45.7)	2 (8.7)
Neuroretinitis	3 (12.5)	1 (4.3)
Central retinal vein occlusion	1 (4.2)	0
Compressive optic neuropathy	1 (4.2)	0
Non-arteritic anterior ischemic optic neuropathy	6 (25)	0

**Figure 1:**
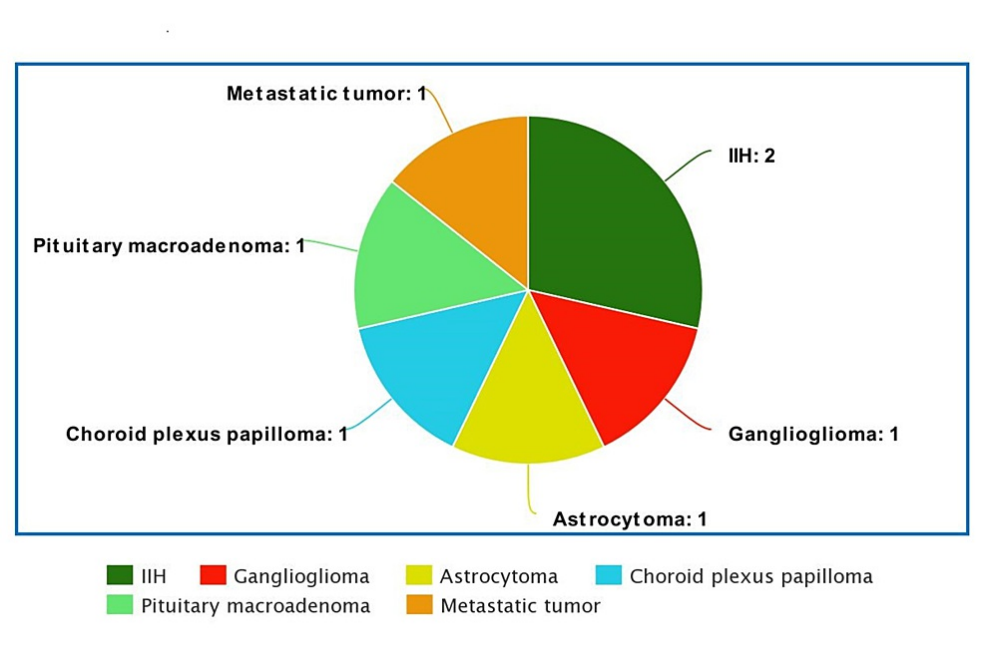
Etiology of papilledema. IIH, idiopathic intracranial hypertension

Among bilateral ODS, the most common cause was papilledema (30.4%), followed by hypertensive retinopathy (21.7%). Other causes of bilateral ODS were diabetic papillitis (13.1%), optic disc drusen (13.1%), infiltrative optic neuropathy (8.7%), ON (8.7%), and neuroretinitis (4.3%) (Table 1). The most common cause of papilledema was a brain tumor (*n* = 5), followed by idiopathic intracranial hypertension (*n* = 2) (Figure 1). There were two ON cases presented as bilateral ODS; however, no NA-AION case was presented as bilateral ODS.

Table 2 displays demographic information and clinical characteristics of unilateral and bilateral ODS. Both unilateral and bilateral ODS were common in those aged over 20 years; however, unilateral ODS cases outnumbered bilateral ODS cases in those aged over 51 years. Both unilateral and bilateral ODS had a similar male-to-female ratio. Females had a higher risk of unilateral and bilateral ODS. Malay was the most common ethnicity for both unilateral and bilateral ODS (*n* = 15 and *n* = 17, respectively), which corresponds to the demographics of our local population. Clinically, 75% of patients with unilateral ODS had a positive relative afferent pupillary defect (RAPD), whereas only 13.1% of patients with bilateral ODS had a positive RAPD. The majority of patients with unilateral and bilateral ODS had reduced vision (87.5% and 69.6%, respectively). Visual field defects were found in 58.3% of unilateral and 34.8% of bilateral ODS cases. Unilateral ODS patients reported more ocular pain (37.5%) than bilateral ODS patients (8.7%).

**Table 2:** Demographic data and clinical characteristics of unilateral and bilateral ODS. ODS, optic disc swelling; RAPD, relative afferent pupillary defect

	Unilateral ODS (*n* = 24)	Bilateral ODS (*n* = 23)
Age group (year), *n* (%)		
≤20	2 (8.3)	3 (13.1)
21-50	10 (41.7)	13 (56.5)
≥50	12 (50.0)	7 (30.4)
Sex (male:female)	8:16	8:15
Race, *n* (%)		
Malay	15 (62.5)	17 (74.0)
Chinese	5 (20.8)	5 (21.7)
Indian	3 (12.5)	1 (4.3)
Others	1 (4.2)	0 (0)
RAPD, *n* (%)		
Present	18 (75.0)	3 (13.1)
Absent	6 (25.0)	20 (86.9)
Vision at presentation, *n* (%)		
6/6	3 (12.5)	7 (30.4)
6/9-6/24	4 (16.7)	4 (17.4)
6/36-6/60	2 (8.3)	3 (13.1)
Worse than 6/60	15 (62.5)	9 (39.1)
Visual field defect, *n* (%)		
Yes	14 (58.3)	8 (34.8)
Enlarged blind spot	4 (16.7)	6 (26.2)
Altitudinal defect	3 (12.5)	1 (4.3)
Others	7 (29.1)	1 (4.3)
No	10 (41.7)	15 (65.2)
Ocular pain, *n* (%)		
Yes	9 (37.5)	2 (8.7)
No	15 (62.5)	21 (91.3)

As shown in Table 3, we compared the demographic data and clinical presentation between NA-AION and ON among the unilateral ODS. The mean age of onset in the NA-AION cases was significantly older than in ON cases (57.5 years vs. 40.3 years, *P* = 0.016). The duration of symptoms was greater in NA-AION cases compared to ON (50.7 ± 29.7 vs. 17.4 ± 25.6; *P* = 0.042). The primary complaint of both NA-AION and ON patients was blurring of vision (*n* = 5 and *n* = 10, respectively), followed by visual field defects (*n* = 1 for each group). Ocular pain was a significant clinical finding in ON cases (54.5%) but not in NA-AION cases (*P* = 0.043). RAPD was present in five NA-AION patients and nine ON patients (*P* = 0.999). There was no significant difference in the initial VA between NA-AION and ON (1.33 ± 0.97 vs. 0.71 ± 0.40). The final VA, however, was significantly improved in ON compared to NA-AION (0.31 ± 0.34 vs. 1.14 ± 1.00; *P* = 0.029).

**Table 3:** Comparison of demographic data and clinical presentation between NA-AION and ON among unilateral ODS. ^#^Mann-Whitney *U* test. ^*^Fisher’s Exact test, *P* < 0.05 is considered statistically significant. NA-AION, non-arteritic anterior ischemic optic neuropathy; ON, optic neuritis; M, male; F, female; RAPD, relative afferent pupillary defect; VA, visual acuity; logMAR, logarithm of the minimum angle of resolution; ODS, optic disc swelling

	NA-AION (*n* = 6)	ON (*n* = 11)	*P*-value
Age (year) (mean)	57.5	40.3	0.016^#^
Sex (M:F)	3:3	3:8	0.600^*^
Duration before the presentation (day)	50.7 ± 29.7	17.4 ± 25.6	0.042^#^
Symptoms at presentation			
Blurring of vision	5	10	0.999^*^
Visual field defect	1	1	0.999^*^
Ocular pain	0	6	0.043^*^
Positive RAPD	5	9	0.999^*^
Initial VA (logMAR)	1.33 ± 0.97	0.71 ± 0.40	0.223^#^
Final VA (logMAR)	1.14 ± 1.00	0.31 ± 0.34	0.029^#^

## Discussion

In our review, we discovered that ON was the most common cause of unilateral ODS, followed by NA-AION. Although NA-AION has been reported as the most common cause of unilateral ODS, this was not the case in our study, most likely because we excluded patients who presented with a pale optic disc, which could be a sequela of NA-AION [2,7]. Arteritic AION was not reported in our study because it is less common than NA-AION, which accounts for 5% to 10% of AION [8]. Aside from ON and NA-AION, Jung et al. reported a small number of unilateral ODS cases in the Korean population due to compressive etiologies, neuroretinitis, pseudopapilledema, optic disc drusen, and papillophlebitis. It was also discovered that the two most common causes of unilateral ODS, NA-AION, and ON have a predilection toward males more than females, with a mean age of 53.4 and 29.2 years, respectively [7].

A local tertiary center reported a similar observation as ours, with the most common cause of unilateral ODS (53%) being ON (46.7%), followed by NA-AION (33.3%). Other etiologies in this study were papilledema (6.7%) and CRVO (13.3%). The majority of their ON cases were females (72%), with a mean age of 39.71 years, but the majority of their NA-AION cases (60%) were males with a mean age of 59.20 years [3].

A study in Turkey also shared similar observations as ours, with a preponderance of unilateral ODS due to ON (48.6%), followed by NA-AION (20%), with a mean age of 40.3 and 64.86 years, respectively. Other unilateral ODS etiologies include retinal vein occlusions, optic disc drusen, sarcoidosis, and idiopathic causes [9].

In this study, papilledema was the most common cause of bilateral ODS, accounting for 30.4%. However, our findings differed from those of Hata and Miyamoto and Iijima et al., who both found that papilledema was responsible for more than 50% of bilateral ODS [2,10]. This could be due to some inpatient cases being referred to the on-call team and thus not being recorded in our clinic data. Of the total patients with bilateral ODS, 13.1% had a positive RAPD because there was a discrepancy in ODS between the right and left eyes. We believed RAPD was positive in these patients due to the asymmetrical involvement of the optic nerve between the right and left eyes.

As compared to data from Hata and Miyamoto and Iijima et al. [2,10], we have a higher proportion of bilateral ODS caused by diabetes mellitus (13.1%) and hypertension (21.7%) because Malaysia has one of the highest incidence rates for both diabetes mellitus and hypertension in Asia [11]. Our population has a relatively lower level of awareness and treatment was suboptimal [12]. Jung et al. reported only one diabetic papillitis (11%) in their bilateral ODS [7]. Other minority causes include ON, NA-AION, and pseudopapilledema [7]. Iijima et al. observed that 5% of their bilateral ODS patients were due to hypertensive retinopathy, and there was no reported case of diabetic papillitis [10]. The majority of their bilateral ODS patients are female (55.37%), with an average age of 38.2 years [10]. Urfalioglu et al. reported, in Turkey, only 4.61% and 3.08% had bilateral ODS due to hypertensive retinopathy and diabetic papillitis, respectively [9]. Their study showed a mean age of 34.2 years with a strong predilection toward females (86.15%). Idiopathic intracranial pressure was their leading cause of bilateral ODS (67.7%). ON, optic disc drusen, cavernous sinus thrombosis, intracranial mass, sarcoidosis, Harada syndrome, and idiopathic causes were among the etiologies [9].

We compared the clinical presentation and prognosis of ON and NA-AION in unilateral ODS cases. The mean age of onset was significantly older in NA-AION cases than in ON cases (57.5 years vs. 40.3 years, *P* = 0.016). NA-AION is more common in the elderly because they are more likely to have other systemic comorbidities related to vascular pathologies, such as hypertension, diabetes mellitus, dyslipidemia, and cardiovascular illnesses that lead to microvasculopathy at the optic disc [3,7,9,13,14]. The duration of symptoms is significantly shorter in ON cases than in NA-AION cases. This happens because ON patients were younger and sought treatment sooner than the elderly in NA-AION cases. There was significant ocular pain in ON cases but none in NA-AION cases. This is due to the anatomical location of the trigeminal nerve fibers, which are confined in the optic nerve sheath and located in very close proximity to the superior and medical recti at the orbital apex. Movement of the eye causes traction on the optic nerve sheath, thereby eliciting pain, as hypothesized by Whitnall [15]. In a study by Lepore, ocular pain occurred significantly more often with retrobulbar optic neuropathy. The eye pain does not reflect the severity or origin of optic neuropathy [16]. RAPD was positive in five NA-AION patients and nine ON patients; thus, it was not a significant marker for distinguishing ON from NA-AION. At the first presentation, there was no significant difference in the optic disc features between ON and NA-AION. The ON cases had a better prognosis of final VA than those in NA-AION. NA-AION has a worse visual prognosis due to insufficient blood supply to the optic disc, resulting in an irreversible change in the optic discs [3,7]. The good visual recovery of ON is comparable to that of ONTT and Jung et al. [4,7].

The etiology of ON is primarily idiopathic; however, ON may be related to other causes such as demyelinating disease, autoimmune disease, infectious and parainfectious causes, and so on [17]. According to Ismail et al., more than half of ON cases are idiopathic. There were more cases of neuromyelitis optica (NMO) among their ON patients than multiple sclerosis [18]. Due to some limitations, our study did not address the causes of ON. Multiple sclerosis, NMO, myelin oligodendrocyte glycoprotein (MOG) antibody disease, and other demyelinating diseases are frequently associated with ON. These diseases require additional laboratory tests, such as serum NMO-IgG and serum MOG-IgG, as well as radiological tests, such as magnetic resonance imaging (MRI) [17]. Serum NMO-IgG and MOG-IgG are not routinely tested in our setting because these tests are not readily available.

There are several limitations to this study. Our study was retrospective, and we had to exclude patients who were lost to follow-up. We had to limit our study to the pre-COVID era, as many patients were lost to follow-up between 2020 and 2021 during the COVID-19 outbreak. We sampled our study population from our eye clinic; thus, patients who were referred from the emergency department or inpatient wards might have been missed. Our sample size was limited as it was conducted at one institution. Hence, a data bank should be established soon with the involvement of multidisciplinary teams and multicenter to recruit more patients with ODS and better understand the etiologies of ODS in our population.

## Conclusions

There are many differential diagnoses to be considered when a patient is presented with ODS. A detailed history taking and thorough eye examination should be performed, along with other supportive investigations as indicated, such as visual field, B-scan, computed tomography (CT), or MRI. In our study, the two most common causes of unilateral ODS were ON and NA-AION. When compared to NA-AION patients, ON patients are younger, present earlier, and have better visual recovery. The most common cause of bilateral ODS was papilledema, followed by hypertensive retinopathy and diabetic papillitis. Given the increased prevalence of hypertension and diabetes in Malaysia, it is not surprising that hypertensive retinopathy and diabetic papillitis accounted for one-third of the bilateral ODS patients, a finding not seen in previous studies. This study applies to our population and can serve as guidance when approaching ODS patients in our local setting.

## References

[1] Van Stavern GP (2007). Optic disc edema. Semin Neurol.

[2] Hata M, Miyamoto K (2017). Causes and prognosis of unilateral and bilateral optic disc swelling. Neuroophthalmology.

[3] Abdul Aziz AM, Ismail AS, Yaakub A (2022). The clinical profile of unilateral and bilateral optic disc swelling in a tertiary center in northern Malaysia. Cureus.

[4] (1991). The clinical profile of optic neuritis. Experience of the Optic Neuritis Treatment Trial. Optic Neuritis Study Group. Arch Ophthalmol.

[5] IONDT Research Group (1998). The ischemic optic neuropathy decompression trial (IONDT): design and methods. Control Clin Trials.

[6] Regillo CD, Brown GC, Savino PJ, Byrnes GA, Benson WE, Tasman WS, Sergott RC (1995). Diabetic papillopathy. Patient characteristics and fundus findings. Arch Ophthalmol.

[7] Jung JJ, Baek SH, Kim US (2011). Analysis of the causes of optic disc swelling. Korean J Ophthalmol.

[8] Hayreh SS (2009). Ischemic optic neuropathy. Prog Retin Eye Res.

[9] Urfalioglu S, Ozdemir G, Guler M, Duman GG (2021). The evaluation of patients with optic disc edema: a retrospective study. North Clin Istanb.

[10] Iijima K, Shimizu K, Ichibe Y (2014). A study of the causes of bilateral optic disc swelling in Japanese patients. Clin Ophthalmol.

[11] Abdul-Razak S, Daher AM, Ramli AS (2016). Prevalence, awareness, treatment, control and socio demographic determinants of hypertension in Malaysian adults. BMC Public Health.

[12] Rampal S, Rampal L, Rahmat R, Zain AM, Yap YG, Mohamed M, Taha M (2010). Variation in the prevalence, awareness, and control of diabetes in a multiethnic population: a nationwide population study in Malaysia. Asia Pac J Public Health.

[13] Balducci N, Morara M, Veronese C (2017). Optical coherence tomography angiography in acute arteritic and non-arteritic anterior ischemic optic neuropathy. Graefes Arch Clin Exp Ophthalmol.

[14] Rougier MB, Delyfer MN, Korobelnik JF (2017). OCT angiography of acute non-arteritic anterior ischemic optic neuropathy. J Fr Ophtalmol.

[15] Whitnall SE (1921). The Anatomy of the Human Orbit and Accessory Organs of Vision. London: Henry Frowde and Hodder and Stoughton.

[16] Lepore FE (1991). The origin of pain in optic neuritis. Determinants of pain in 101 eyes with optic neuritis. Arch Neurol.

[17] Hoorbakht H, Bagherkashi F (2012). Optic neuritis, its differential diagnosis and management. Open Ophthalmol J.

[18] Ismail S, Wan Hazabbah WH, Muhd-Nor NI, Daud J, Embong Z (2012). Clinical profile and aetiology of optic neuritis in Hospital Universiti Sains Malaysia--5 years review. Med J Malaysia.

